# Supplementary material for: Evaluating Shared Decision Making in Trial of Labor After Cesarean Counseling Using Objective Structured Clinical Examinations
Source: MedEdPORTAL. 2020 Mar 20;16:10891. doi: 10.15766/mep_2374-8265.10891 (PMC7182044; doi:10.15766/mep_2374-8265.10891)
Supplement: Supplementary file 1 — A. Case 1 SP Development Tool.docx B. Case 2 SP Development Tool.docx C. Case 3 SP Development Tool.docx D. Case 1 Door Note.docx E. Case 2 Door Note.docx F. Case 3 Door Note.docx G. Scoring Rubric.docx [file mep-16-10891-s001.zip › _Educational_Summary_Report_10891.pdf]

MedEdPORTAL® The AAMC Journal of Teaching and Learning Resources

Original Publication Open Access

Evaluating Shared Decision Making in Trial of Labor After Cesarean Counseling Using Objective Structured Clinical Examinations

Brownsyne Tucker Edmonds, MD, MPH, MS*, Shelley M. Hoffman, MPH, Tatiana Laitano, MD, Fatima McKenzie, MS, Janet Panoch, MA, Abigail Litwiller, MD, Mark J. Di Corcia, PhD, OTR

*Corresponding author: btuckere@iupui.edu

Abstract

Introduction: Although shared decision making (SDM) is optimal for trial of labor after cesarean (TOLAC) counseling, resources to assess residents' clinical competency and communication skills are lacking. We addressed this gap by developing and testing an objective structured clinical examination (OSCE) to evaluate whether learners were able to use SDM in TOLAC counseling. **Methods:** We created three simulation scenarios with increasing complexity to assess the skills of residents in their first, second, or third postgraduate year in using SDM in TOLAC counseling. All cases involved a standardized patient requesting a TOLAC consultation. Residents were provided with a medical history and instructed to counsel and develop a care plan. A 10-item scoring rubric was used, and each item was rated 0 (absent), 1 (partial), or 2 (complete). Three coders independently rated the encounters; discrepancies were resolved by consensus. **Results:** Over 3 years, 39 residents participated in 60 OSCE encounters. The majority provided complete discussions of the clinical issue (93%), chances of success (72%), and maternal and fetal risks (100% and 85%, respectively) but obtained partial assessments of understanding (78%). Discussions of benefits were typically absent, with the exception of the maternal benefits (47%). More than 40% of residents did not discuss the patient's goals, 53% lacked discussion of uncertainties related to TOLAC, and half failed to explore the patient's preference, with most deferring a decision to a future encounter. **Discussion:** Residents consistently discussed diagnosis, prognosis, and maternal risks yet infrequently addressed goals and preferences—two critical elements of SDM.

Keywords
TOLAC, Trial of Labor, VBAC, Vaginal Birth After Cesarean, Shared Decision Making, Birth Counseling, Resident Training, Communication Skills, OB/GYN, Case-Based Learning, Clinical Skills Assessment/OSCEs, Simulation, Standardized Patient

Educational Objectives

By the end of this objective structured clinical examination, learners will be able to:

1. Elicit an appropriate history to individualize the patient's risk assessment (PGY 1-3).
2. Convey accurate estimates and/or descriptions of risk and chance of success (PGY 1-3).
3. Exhibit shared decision-making behaviors, including values elicitation (PGY 1-3).
4. Respectfully negotiate (possible) conflict of physician recommendation with patient's wishes (PGY 3 only).

Citation:
Tucker Edmonds B, Hoffman SM, Laitano T, et al. Evaluating shared decision making in trial of labor after cesarean counseling using objective structured clinical examinations. *MedEdPORTAL*. 2020;16:10891. https://doi.org/10.15766/mep_2374-8265.10891

Introduction

Pregnant women with a history of a prior cesarean birth may be offered the option to either attempt a trial of labor after cesarean (TOLAC), with the goal of achieving a vaginal birth after cesarean (VBAC), or schedule an elective repeat cesarean delivery (ERCD). Studies have found that women who achieve a successful VBAC experience fewer complications and shorter recovery periods.[1,2] Additionally, VBAC may be a good option for those who want to have multiple pregnancies in the future, as multiple cesarean deliveries can lead to additional complications at the time of birth, including an unplanned hysterectomy, infection, and abnormal placentation.[3,4] Nevertheless, TOLAC is not without risks. Due to the presence of the surgical scar on the uterine wall, women who attempt TOLAC are at increased risk of rupturing the uterine wall, which can lead to life-threatening hemorrhage, fetal demise, and/or emergency repeat cesarean delivery (RCD).[5-7] Such events are rare but potentially catastrophic. Furthermore, even if not emergent, unscheduled cesarean deliveries that occur in the

Copyright © 2020 Tucker Edmonds et al. This is an open-access publication distributed under the terms of the Creative Commons Attribution license.

wake of a failed TOLAC (due to arrested labor or nonreassuring fetal heart tracings) result in greater morbidity (e.g., infection, blood loss) than a scheduled RCD.[8] Considering that women will value these risks and benefits differently and due to the uncertainties surrounding the outcome of TOLAC, the decision for TOLAC versus RCD can be a highly preference-sensitive decision.

Preference-sensitive decision making is best deliberated by way of a shared decision-making (SDM) model of communication.[9] SDM is a collaborative, bidirectional model of communication wherein providers not only inform patients of risks, benefits, and prognosis but also elicit patients' values and negotiate competing priorities in the process of making a joint decision regarding treatment or clinical care.[10,11] The SDM model is increasingly promoted as the optimal framework for patient and physician engagement in clinical care. Therefore, now more than ever, it is critical for the provider and patient to learn to engage in an SDM process about the available birth options—a process that not only considers the risks and benefits of each alternative but also examines the patient's preferences and goals to achieve an optimally informed decision. Research has shown that although patients value providers' knowledge and guidance, women report a higher level of satisfaction with their birthing experiences when they are directly involved in the decision making.[9,12] Thus, the American College of Obstetricians and Gynecologists (ACOG) supports the SDM approach when counseling women for TOLAC, as detailed in the 2010 Practice Bulletin.[13]

In light of these developments in clinical practice, the most recent edition of the Accreditation Council for Graduate Medical Education's obstetrics and gynecology milestone assessment now requires residents to exhibit competency in SDM as a goal for completion by graduation.[14,15] Currently, there are no tools that we could find in *MedEdPORTAL* or other publications to evaluate and train residents in SDM in the context of TOLAC counseling. Objective structured clinical examinations (OSCEs) are widely used in residency curriculums to assess resident clinical competency and communication skills. They serve as a promising tool to observe and assess SDM and other patient-centered communication skills in learners. Our assessment activity sought to address this gap by developing and pilot testing an OSCE to evaluate whether learners were able to use SDM in TOLAC counseling.

Methods

Learner Population

We created three simulation-based scenarios to assess obstetrics and gynecology physician residents' skills in the use of SDM in TOLAC counseling between 2013 and 2015 at Indiana University School of Medicine. Although all residents were required to participate in the examination, inclusion of their examination results was voluntary. The OSCE was conducted in a controlled environment at the university's simulation center and monitored by our team. Residents did not require any equipment for this simulation and did not have any prereading assignments. The OSCE served as an assessment of skills and content that they were exposed to during the course of clinical care and didactic lectures. Within the curriculum, residents received one didactic lecture on SDM and were trained in TOLAC counseling as early as the first year in residency. Additionally, we did not have to recruit any standardized patients (SPs), as the university had an established simulation center that provided the necessary staff, as well as trained and experienced SPs, to conduct these types of training sessions. For this OSCE, we provided a trained SP with the SP Case Development Tool for her to learn to portray Brenda Washington on the day of examination. This SP Case Development Tool provides enough information for any volunteering faculty or staff member, or even a student, to portray the patient and successfully carry out this OSCE in settings that do not have access to trained SPs and actors. The assessment was approved as exempt level by the Indiana University Institutional Review Board (IRB# 1304011110). OSCEs are standard practice in medical education and are routinely video and audio recorded, regardless of research intention. No additional information was provided to residents regarding this assessment because they were not asked to do anything beyond what was routinely required for the OSCE testing conducted as a part of their residency education, assessment, and evaluation.

Case Development

When we first developed this OSCE, we created only one case (Case 1), and thus, in 2013, residents of all PGY levels completed OSCE Case 1. The next year, we developed two more cases (Cases 2 and 3), each one increasing in clinical complexity and therefore decreasing the likelihood of successful VBAC, in an effort to challenge upper-level residents. For the 2014 OSCE assessment, residents were presented with cases that corresponded to their residency level (e.g., PGY 1 = Case 1, PGY 2 = Case 2, PGY 3 = Case 3). Only PGY 2 (Case 2) was evaluated in 2015. New content was introduced for the PGY 1 examination that year. Residents completed only one TOLAC OSCE case each year, and due to the timing for which we initiated this OSCE assessment, PGY 1 residents in 2013 were the only cohort tested on all three cases from 2013 to 2015. Because each OSCE case was conducted on different dates

Copyright © 2020 Tucker Edmonds et al. This is an open-access publication distributed under the terms of the Creative Commons Attribution license.

and at different times, only one SP was needed to implement this assessment.

All three cases depicted an African American woman presenting at 35 + 4 weeks' gestation to the outpatient clinic and requesting a TOLAC consultation. The patient's age, obstetrical history, risk factors, social history, and preferences and priorities varied in each case. As an employee of the university's simulation center, the SP did not require any additional training to help conduct this simulation but was provided with detailed instructions to portray specific symptoms and psychosocial characteristics for each case (for instructions, see Appendix A for Case 1, Appendix B for Case 2, and Appendix C for Case 3). The SP was provided with a backstory of relevant values, goals, and preferences, which she was instructed to reveal only if and when prompted by the resident, reflecting priorities such as safety concerns, recovery time, childcare support, birth experience, and future fertility. The psychosocial profile was meant to reflect a patient with mixed feelings regarding VBAC. The SP was instructed to offer information regarding her goals and preferences to the resident only if the resident elicited this information, considering that values clarification and preference elicitation were SDM skills that were being evaluated. Uniformly, the SP was permitted to express that her primary goal/value was to do "whatever was safest for her and her baby." Therefore, if the resident suggested that one course of action was safer than the other, the patient would accept this as true and defer to the physician's recommendation. These rules of engagement were standardized for all encounters. The story was developed to convey a complex family situation with limited support and the need to care for three children. The SP was to convey having the impression that VBAC could be dangerous, based on a coworker's experience. Furthermore, the idea of a fixed date for a scheduled cesarean delivery would be very convenient for her with regard to arranging childcare. However, the lengthy recovery would be suboptimal given her childcare commitments and lack of family support. Overall, despite these concerns, she was open to doing whatever would be safest for her and her baby.

Trainee Instructions

Prior to the encounter, resident physicians reviewed a door note designed to act as the SP's medical record. The note detailed the patient's medical history, vitals, and examination findings. Each note varied slightly based on each case (for door notes, see Appendix D for Case 1, Appendix E for Case 2, and Appendix F for Case 3). Sufficient information was provided to allow residents to calculate an estimate of VBAC success using the available online calculator if they chose to do so, but this was not required or suggested. We did not capture whether the VBAC calculator was utilized because the residents were neither rated more highly for utilizing the calculator nor penalized for failing to do so. They were simply permitted to do so if this was a part of their usual approach to counseling. The residents were given up to 18 minutes to review the door note, counsel the SP (maximum: 15 minutes), and document their discussion. Notably, the residents were not aware that SDM was being assessed at the station. The residents received a debrief and feedback after completing their examination. Results were collected by both the OSCE developing team and the program directors and included in the residents' portfolios. No residents scored poorly enough on the overall assessment to require remediation.

Coding

A 10-item scoring rubric was used to assess the quality of SDM in the TOLAC consultations and was adapted from Braddock's previously developed nine-item informed decision-making scale (see Appendix G for the scoring rubric).[16-18] Specifically, this rubric was used to evaluate the resident's discussion of the following elements: nature of the clinical issue and prognosis, alternative options (i.e., TOLAC, TOLAC with possibility of repeat cesarean, or an ERCD), and associated risks and benefits for the mother and baby. Additionally, residents were also scored for eliciting the patient's preferences and goals for birth. Encounters were scored by three authors (Brownsyne Tucker Edmonds, Fatima McKenzie, and Janet Panoch). Discussions were scored for completeness and rated as 0 (absent), 1 (partial), or 2 (complete). For example, when the resident had to assess the patient's understanding, for a "partial" scoring the resident had to inquire as to whether the patient understood ("Do you have any questions about the options we discussed?"), whereas for a "complete" score the resident was required to ask the patient to demonstrate understanding (teach-back required by the patient). Coders independently scored each encounter at the same time during the examination, and any discrepancies in scores were discussed after each encounter to reach consensus. Encounters were audio and video recorded, as well as transcribed for analysis. Descriptive statistics were produced using SPSS version 24.

Results

Learner Population

Sixty OSCE examinations took place between 2013 and 2015 (PGY 1 = 15, PGY 2 = 28, PGY 3 = 17). Taking those examinations were 39 residents, 21 of whom participated in more than one OSCE due to their progression in residency. For example, residents in 2013 who were tested on Case 1 were

Copyright © 2020 Tucker Edmonds et al. This is an open-access publication distributed under the terms of the Creative Commons Attribution license.

also tested on Case 2 in 2014 and Case 3 in 2015. The majority of residents were female, roughly two-thirds were white, and the average age was 30 years. Case 1 was completed by the most residents ($n = 34$) compared to Cases 2 and 3 ($ns = 19$ and 7, respectively). Table 1 lists the case presentations for each year and the number of residents who participated in each case.

Rubric Scoring

In all cases, the majority of residents consistently scored "complete" for discussing the clinical nature of TOLAC, informing the patient of her chance at having a successful VBAC, and presenting her with alternatives (see Table 2). More than 80% of residents addressed the fetal/neonatal risks specific to TOLAC but did not mention the fetal/neonatal and maternal benefits of any birth option. All residents scored "complete" for discussing the maternal risks of TOLAC. Although 62% of residents in Case 1 discussed the maternal risks of scheduling a repeat cesarean birth, fewer than half did the same in Case 3, followed by 16% in Case 2.

The majority of residents scored "absent" on two SDM items: eliciting the patient's preference on the basis of the conversation (Case 2 = 53%, Case 3 = 71%) and discussing the associated uncertainties of TOLAC (Case 2 = 69%). Additionally, partial credit was primarily given for assessing the patient's understanding (Case 1 = 91%, Case 2 = 63%, Case 3 = 57%). In particular, residents were noted to typically ask "Do you have any questions?" or "What questions do you have for me?" without utilizing teach-back methods or explicitly inquiring about or evaluating the patient's ability to explain in her own words her understanding of what had been discussed. Although residents received variable scores in Cases 1 and 2 for eliciting the patient's goals, desired birth experience, and competing priorities, nearly three-quarters of upper-level residents in Case 3 completed this task.

A similar trend can be seen in the Figure, where we compared "complete" scores in Case 1 by residency year (PGY 1 = 15, PGY 2 = 9, PGY 3 = 10). All residents scored "complete" for discussing the clinical issue, the alternative modes of delivery, and the risks of TOLAC to the mother. In contrast, 20% or fewer completely discussed the fetal benefits for each mode of delivery and the maternal benefit of scheduling a TOLAC (with the possibility of emergency repeat cesarean). Likewise, only 9% assessed the patient's understanding of the discussion.

Table 1. Total Case Presentations by Year and Residency Level ($N = 60$)[a]

Residency Level	2013: Case 1	2014 Case 1	2014 Case 2	2014 Case 3	2015: Case 2
PGY 1	6	9			
PGY 2	9		9		10
PGY 3	10			7	

[a]Not all cases were available each year.

We also compared "complete" scores across residency level, regardless of the OSCE case assigned. Residents scored similarly for rubric items that pertained to discussing the clinical nature of the situation (Table 3). When discussing elements of SDM, more PGY 1 residents received "complete" scores for discussing the patient's role in the decision-making process (73%) and exploring the patient's preference (53%) compared to the second-year (21% and 14%, respectively) and third-year residents (47% and 18%, respectively).

Discussion

We set out to develop and test an OSCE to evaluate the use of SDM in TOLAC counseling by obstetrics and gynecology residents. We found that residents consistently addressed informed consent components, such as discussions of diagnosis, prognosis, and maternal risk, in their counseling. However, they infrequently addressed maternal and neonatal benefits, uncertainties, or the patient's goals or preferences. This suggests that critical elements of SDM are absent and that interventions and training are needed for improvement. We developed the cases with increasing degrees of complexity. Case 1 was intended to represent a patient with a high likelihood of successful VBAC; Case 2, for second-years, with lower likelihood; and Case 3, for third-years, with greater risks related to having had two prior cesarean deliveries. We were surprised at the number of third-years who were comfortable with the concept of a TOLAC for a patient with two prior cesarean deliveries. We had expected the residents to recommend against TOLAC and planned to evaluate how well they negotiated potentially conflicting opinions with the patient to reach an agreed-upon plan of care. Instead, many residents expressed concern to the patient that their attending physicians might not be comfortable but that, as supported by the ACOG Practice Bulletin,[13] a patient with up to two prior cesareans was an appropriate candidate. This may mark a noteworthy shift, generationally, in attitudes toward TOLAC that warrants further exploration.

Furthermore, we were also surprised that the PGY 3 scores were lower than the PGY 1 and PGY 2 scores. One might assume that as their training progressed, residents would become more skilled or competent in these domains such that the PGY 3

Copyright © 2020 Tucker Edmonds et al. This is an open-access publication distributed under the terms of the Creative Commons Attribution license.

Table 2. TOLAC OSCE Evaluation Scores by Rubric Element and Case

	Case 1 (*N* = 34)			Case 2 (*N* = 19)			Case 3 (*N* = 7)		
**Element**	**No. (%) Complete**	**No. (%) Partial**	**No. (%) Absent**	**No. (%) Complete**	**No. (%) Partial**	**No. (%) Absent**	**No. (%) Complete**	**No. (%) Partial**	**No. (%) Absent**
Discussion of clinical issue	34 (100)	0 (0)	0 (0)	15 (79)	4 (21)	0 (0)	7 (100)	0 (0)	0 (0)
Discussion of prognosis	20 (59)	11 (32)	3 (9)	18 (95)	1 (5)	0 (0)	5 (71)	1 (14)	1 (14)
Discussion of alternatives									
TOLAC	34 (100)	0 (0)	0 (0)	18 (95)	1 (5)	0 (0)	7 (100)	0 (0)	0 (0)
TOLAC + RCD[a]	31 (91)	3 (9)	0 (0)	9 (47)	6 (32)	4 (21)	5 (71)	2 (29)	0 (0)
Scheduled ERCD[b]	32 (94)	0 (0)	2 (6)	19 (100)	0 (0)	0 (0)	5 (71)	1 (14)	1 (14)
Discussion of fetal risks									
TOLAC	29 (85)	2 (6)	3 (9)	16 (84)	1 (5)	2 (10)	6 (86)	1 (14)	0 (0)
TOLAC + RCD[a]	20 (59)	7 (21)	7 (21)	1 (5)	3 (16)	15 (79)	0 (0)	1 (14)	6 (86)
Scheduled ERCD[b]	14 (41)	0 (0)	20 (59)	3 (16)	1 (5)	15 (79)	0 (0)	0 (0)	7 (100)
Discussion of maternal risks									
TOLAC	34 (100)	0 (0)	0 (0)	19 (100)	0 (0)	0 (0)	7 (100)	0 (0)	0 (0)
TOLAC + RCD[a]	29 (85)	0 (0)	5 (15)	11 (58)	1 (5)	7 (37)	3 (43)	1 (14)	3 (43)
Scheduled ERCD[b]	21 (62)	8 (23)	5 (15)	3 (16)	5 (26)	11 (58)	3 (43)	2 (29)	2 (29)
Discussion of fetal benefits									
TOLAC	5 (15)	0 (0)	29 (85)	3 (16)	0 (0)	16 (84)	1 (14)	0 (0)	6 (86)
TOLAC + RCD[a]	0 (0)	0 (0)	34 (100)	0 (0)	0 (0)	19 (100)	0 (0)	0 (0)	7 (100)
Scheduled ERCD[b]	1 (3)	0 (0)	33 (97)	1 (5)	0 (0)	18 (95)	0 (0)	1 (14)	6 (86)
Discussion of maternal benefits									
TOLAC	17 (50)	3 (9)	14 (41)	7 (37)	1 (5)	11 (58)	4 (57)	1 (14)	2 (29)
TOLAC + RCD[a]	0 (0)	0 (0)	34 (100)	0 (0)	0 (0)	19 (100)	0 (0)	0 (0)	7 (100)
Scheduled ERCD[b]	13 (38)	2 (6)	19 (56)	5 (26)	1 (5)	13 (68)	2 (29)	2 (29)	3 (43)
Discussion of patient's goals	8 (23)	10 (29)	16 (47)	4 (21)	7 (37)	8 (42)	5 (71)	1 (14)	1 (14)
Discussion of uncertainties	13 (38)	4 (12)	17 (50)	2 (10)	4 (21)	13 (68)	3 (43)	2 (29)	2 (29)
Discussion of patient's role in decision making	15 (44)	9 (26)	10 (29)	6 (32)	4 (21)	9 (47)	4 (57)	3 (43)	0 (0)
Assessment of patient's understanding	3 (9)	31 (91)	0 (0)	6 (32)	12 (63)	1 (5)	3 (43)	4 (57)	0 (0)
Discussion of patient's preference	10 (29)	9 (26)	15 (44)	3 (16)	6 (32)	10 (53)	2 (29)	0 (0)	5 (71)

Abbreviations: ERCD, elective repeat cesarean delivery; OSCE, objective structured clinical examination; RCD, repeat cesarean delivery; TOLAC, trial of labor after cesarean.
[a]RCD was required after an unsuccessful TOLAC.
[b]Cesarean delivery was opted for in lieu of TOLAC.

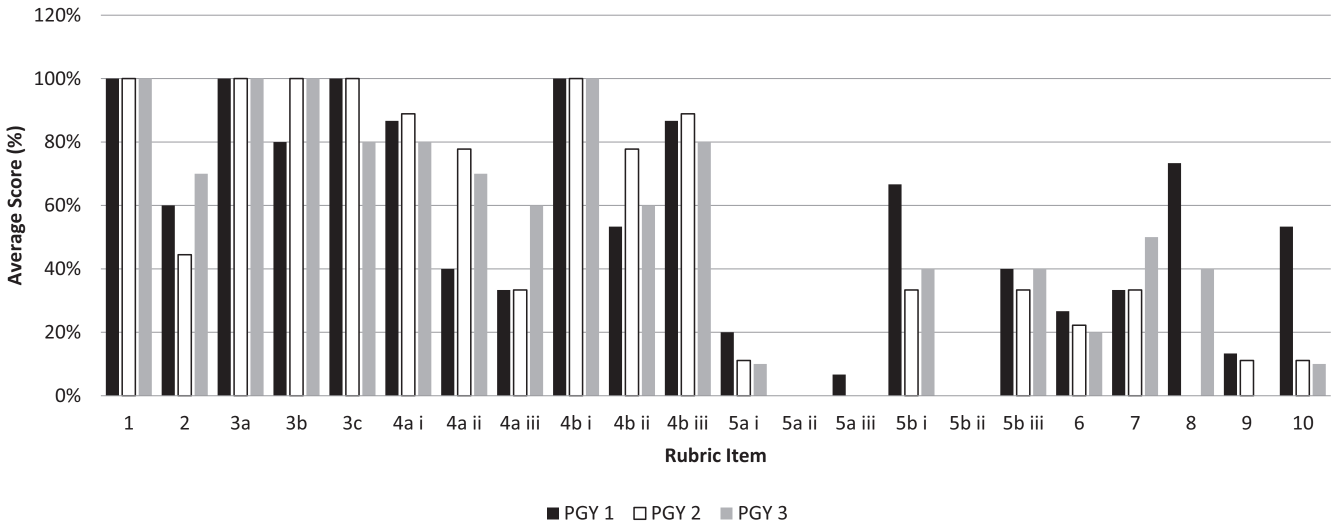

Figure. Percentage of residents (PGY 1 n = 15, PGY 2 n = 9, PGY 3 n = 10) who received a score of "complete" for each rubric item in Case 1 of the trial of labor after cesarean counseling objective structured clinical examination.

Copyright © 2020 Tucker Edmonds et al. This is an open-access publication distributed under the terms of the Creative Commons Attribution license.

Table 3. Scores of “Complete” for Each Rubric Item by Residency Level

Item	PGY 1 (*N* = 15): No. (%)	PGY 2 (*N* = 28): No. (%)	PGY 3 (*N* = 17): No. (%)
Discussion of clinical issue	15 (100)	24 (86)	17 (100)
Discussion of prognosis	9 (60)	22 (79)	12 (71)
Discussion of alternatives			
TOLAC	15 (100)	27 (96)	17 (100)
TOLAC + RCD[a]	12 (80)	18 (64)	15 (88)
Scheduled ERCD[b]	15 (100)	28 (100)	13 (76)
Discussion of fetal risks			
TOLAC	13 (87)	24 (86)	14 (82)
TOLAC + RCD[a]	6 (40)	8 (29)	7 (41)
Scheduled ERCD[b]	5 (33)	6 (21)	6 (35)
Discussion of maternal risks			
TOLAC	15 (100)	28 (100)	17 (100)
TOLAC + RCD[a]	8 (53)	10 (36)	9 (53)
Scheduled ERCD[b]	13 (87)	19 (68)	11 (65)
Discussion of fetal benefits			
TOLAC	3 (20)	4 (14)	2 (12)
TOLAC + RCD[a]	0 (0)	0 (0)	0 (0)
Scheduled ERCD[b]	1 (7)	1 (4)	0 (0)
Discussion of maternal benefits			
TOLAC	10 (67)	10 (36)	8 (47)
TOLAC + RCD[a]	0 (0)	0 (0)	0 (0)
Scheduled ERCD[b]	6 (40)	8 (29)	6 (35)
Discussion of patient’s goals	4 (27)	6 (21)	7 (41)
Discussion of uncertainties	5 (33)	5 (18)	8 (47)
Discussion of patient’s role in decision making	11 (73)	6 (21)	8 (47)
Assessment of patient’s understanding	2 (13)	7 (25)	3 (18)
Discussion of patient’s preference	8 (53)	4 (14)	3 (18)

Abbreviations: ERCD, elective repeat cesarean delivery; RCD, repeat cesarean delivery; TOLAC, trial of labor after cesarean.
[a]RCD was required after an unsuccessful TOLAC.
[b]Cesarean delivery was opted for in lieu of TOLAC.

residents would have higher “complete” scores across the board as compared to residents in PGY 1 and PGY 2. However, it is possible over the course of training that a provider becomes more accustomed to providing certain routine aspects or topics of counseling and develops a scripted approach to counseling that may be less patient centered and individualized. We did not see a clear trend by year to support this postulation, but it provides one potential explanation worth consideration.

There are important limitations to consider in interpreting our findings. Because this was an assessment performed among residents in a single academic medical center, the generalizability of our findings is limited. However, we do believe that selection bias was mitigated, as all residents were required to participate in the annual OSCEs. Although our SP role was developed to be portrayed by either a professional SP or a volunteer, we are aware that this study was made possible in part due to the in-house resources our university’s simulation center provided. For example, the testing room was set up to resemble an outpatient clinic room and had audiovisual recording capability such that encounters could be observed and scored in real time from a control room. Additionally, the center utilized professional SPs, which helped to optimize the success of this simulation, as they were able to act or portray a range of patient needs, concerns, and emotions, all of which enhanced the examination to feel more realistic and applicable to the encounters that residents would face in the clinical setting. Furthermore, Hawthorne effects and social desirability biases are of particular concern in simulation-based studies. In an attempt to diminish social desirability bias, we neither informed residents of our assessment ahead of time nor indicated that their objective was to utilize SDM to counsel the patient. That said, we recognize that because the encounters were recorded as part of an examination, residents’ performance may not have accurately represented their actual counseling behaviors and/or content in practice. It is possible that residents may have attempted to put their best foot forward, resulting in overestimations of their SDM behaviors. Conversely, considering that they were not specifically instructed to use an SDM approach, they may have assumed that they were being tested on content, which may have biased their behaviors toward discussion of more informational content to show what they knew.

Conclusion

This resource makes an important and needed contribution to the education and counseling literature. As SDM becomes

Copyright © 2020 Tucker Edmonds et al. This is an open-access publication distributed under the terms of the Creative Commons Attribution license.

more widely espoused as the optimal model for patient-provider decision making in preference-sensitive care, tools will be needed to ensure that trainees have mastered the skills and achieved competency in this area. Identifying preference-sensitive clinical decisions and utilizing OSCE testing constitute a useful strategy to evaluate and train residents in both basic and advanced communication skills to advance the provision of patient-centered care. Didactic lectures provide a baseline set of content and knowledge; however, building skills in advanced communication techniques requires practice and rehearsal. Observed interactions in simulated settings with SPs or actors trained in improvisation would likely lend themselves best to practicing SDM skills and techniques.

Appendices

A. Case 1 SP Development Tool.docx
B. Case 2 SP Development Tool.docx
C. Case 3 SP Development Tool.docx
D. Case 1 Door Note.docx
E. Case 2 Door Note.docx
F. Case 3 Door Note.docx
G. Scoring Rubric.docx

All appendices are peer reviewed as integral parts of the Original Publication.

Brownsyne Tucker Edmonds, MD, MPH, MS: Associate Professor, Department of Obstetrics and Gynecology, Indiana University School of Medicine; Assistant Dean for Diversity Affairs, Indiana University School of Medicine

Shelley M. Hoffman, MPH: Research Coordinator, Department of Obstetrics and Gynecology, Indiana University School of Medicine

Tatiana Laitano, MD: Research Assistant, Department of Obstetrics and Gynecology, Indiana University School of Medicine

Fatima McKenzie, MS: Research Coordinator, Department of Obstetrics and Gynecology, Indiana University School of Medicine

Janet Panoch, MA: Research Assistant, Department of Obstetrics and Gynecology, Indiana University School of Medicine

Abigail Litwiller, MD: Associate Professor of Clinical Obstetrics and Gynecology, Department of Obstetrics and Gynecology, University of Illinois College of Medicine; Associate Department Head for Education and Faculty Development, Department of Obstetrics and Gynecology, University of Illinois College of Medicine; Residency Program Director, Department of Obstetrics and Gynecology, University of Illinois College of Medicine

Mark J. Di Corcia, PhD, OTR: Assistant Dean for Medical Education and Academic Affairs, Florida Atlantic University Charles E. Schmidt College of Medicine; Associate Professor of Integrated Medical Science, Florida Atlantic University Charles E. Schmidt College of Medicine

Disclosures
None to report.

Funding/Support
None to report.

Ethical Approval
The Indiana University Institutional Review Board approved this study.

References

1. Macones GA, Peipert J, Nelson DB, et al. Maternal complications with vaginal birth after cesarean delivery: a multicenter study. *Am J Obstet Gynecol*. 2005;193(5):1656-1662. https://doi.org/10.1016/j.ajog.2005.04.002
2. Mercer BM, Gilbert S, Landon MB, et al. Labor outcomes with increasing number of prior vaginal births after cesarean delivery. *Obstet Gynecol*. 2008;111(2):285-291. https://doi.org/10.1097/AOG.0b013e31816102b9
3. Zia S, Rafique M. Intra-operative complications increase with successive number of cesarean sections: myth or fact? *Obstet Gynecol Sci*. 2014;57(3):187-192. https://doi.org/10.5468/ogs.2014.57.3.187
4. Cook JR, Jarvis S, Knight M, Dhanjal MK. Multiple repeat caesarean section in the UK: incidence and consequences to mother and child. A national, prospective, cohort study. *BJOG*. 2013;120(1):85-91. https://doi.org/10.1111/1471-0528.12010
5. Landon MB, Hauth JC, Leveno KJ, et al; for the National Institute of Child Health and Human Development Maternal–Fetal Medicine Units Network. Maternal and perinatal outcomes associated with a trial of labor after prior cesarean delivery. *N Engl J Med*. 2004;351(25):2581-2589. https://doi.org/10.1056/NEJMoa040405
6. Lydon-Rochelle M, Holt VL, Easterling TR, Martin DP. Risk of uterine rupture during labor among women with a prior cesarean delivery. *N Engl J Med*. 2001;345(1):3-8. https://doi.org/10.1056/NEJM200107053450101
7. Smith GCS, Pell JP, Cameron AD, Dobbie R. Risk of perinatal death associated with labor after previous cesarean delivery in uncomplicated term pregnancies. *JAMA*. 2002;287(20):2684-2690. https://doi.org/10.1001/jama.287.20.2684
8. Hibbard JU, Ismail MA, Wang Y, Te C, Karrison T, Ismail MA. Failed vaginal birth after a cesarean section: how risky is it? I. Maternal morbidity. *Am J Obstet Gynecol*.

Copyright © 2020 Tucker Edmonds et al. This is an open-access publication distributed under the terms of the Creative Commons Attribution license.

2001;184(7):1365-1373. https://doi.org/10.1067/mob.2001.115044

9. Nieuwenhuijze MJ, Korstjens I, de Jonge A, de Vries R, Lagro-Janssen A. On speaking terms: a Delphi study on shared decision-making in maternity care. *BMC Pregnancy Childbirth*. 2014;14:223. https://doi.org/10.1186/1471-2393-14-223
10. King A, Hoppe RB. “Best practice” for patient-centered communication: a narrative review. *J Grad Med Educ*. 2013;5(3):385-393. https://doi.org/10.4300/JGME-D-13-00072.1
11. Kaimal AJ, Kuppermann M. Understanding risk, patient and provider preferences, and obstetrical decision making: approach to delivery after cesarean. *Semin Perinatol*. 2010;34(5):331-336. https://doi.org/10.1053/j.semperi.2010.05.006
12. Cook K, Loomis C. The impact of choice and control on women’s childbirth experiences. *J Perinat Educ.* 2012;21(3):158-168. https://doi.org/10.1891/1058-1243.21.3.158
13. ACOG Practice Bulletin no. 115: vaginal birth after previous cesarean delivery. *Obstet Gynecol*. 2010;116(2):450-463. https://doi.org/10.1097/AOG.0b013e3181eeb251
14. Bienstock JL, Edgar L, McAlister R; for the Obstetrics and Gynecology Working Group. Obstetrics and gynecology milestones. *J Grad Med Educ*. 2014; 6(1)(suppl 1):126-128. https://doi.org/10.4300/JGME-06-01s1-08
15. The Obstetrics and Gynecology Milestone Project. *J Grad Med Educ*. 2014;6(1)(suppl 1):129-143. https://doi.org/10.4300/JGME-06-01s1-07
16. Braddock CH III, Edwards KA, Hasenberg NM, Laidley TL, Levinson W. Informed decision making in outpatient practice: time to get back to basics. *JAMA*. 1999;282(24):2313-2320. https://doi.org/10.1001/jama.282.24.2313
17. Braddock C III, Hudak PL, Feldman JJ, Bereknyei S, Frankel RM, Levinson W. “Surgery is certainly one good option”: quality and time-efficiency of informed decision-making in surgery. *J Bone Joint Surg Am*. 2008;90(9):1830-1838. https://doi.org/10.2106/JBJS.G.00840
18. Salyers MP, Matthias MS, Fukui S, et al. A coding system to measure elements of shared decision making during psychiatric visits. *Psychiatr Serv*. 2012;63(8):779-784. https://doi.org/10.1176/appi.ps.201100496

Received: March 21, 2019
Accepted: October 21, 2019
Published: March 20, 2020

Copyright © 2020 Tucker Edmonds et al. This is an open-access publication distributed under the terms of the Creative Commons Attribution license.
