# Supplementary material for: Evaluating Shared Decision Making in Trial of Labor After Cesarean Counseling Using Objective Structured Clinical Examinations
Source: MedEdPORTAL. 2020 Mar 20;16:10891. doi: 10.15766/mep_2374-8265.10891 (PMC7182044; doi:10.15766/mep_2374-8265.10891)
Supplement: Supplementary file 1 — A. Case 1 SP Development Tool.docx B. Case 2 SP Development Tool.docx C. Case 3 SP Development Tool.docx D. Case 1 Door Note.docx E. Case 2 Door Note.docx F. Case 3 Door Note.docx G. Scoring Rubric.docx [file mep-16-10891-s001.zip › B. Case 2 SP Development Tool.docx]

Appendix B: *MedEdPORTAL* Standardized Patient Case Development Tool

Date: 2013-2015 (academic year)

Primary Case Author: Brownsyne Tucker Edmonds, MD, MPH, MS

Secondary Case Author: N/A

Standardized Patient Educator: Not available – Our University’s simulation center employs trained standardized patients who provide support for all simulation training efforts conducted.

Name of Case: TOLAC OSCE Counseling Case 2

Name of educational and or assessment activity: TOLAC OSCE Counseling

Patient Name: Brenda Washington

Chief Complaint: Referred for VBAC consultation

Most likely Diagnosis and Differential with rationale from history and/or physical exam: N/A

Challenge question: N/A

Domains: Check all that apply

- Professionalism

X Communication and Interpersonal skills

- Medical History
- Physical exam

X Shared Decision Making

- Patient Education
- Clinical Reasoning
- Documentation
- Handoff
- Presentation
- Other:

Type and level of learner: Case 2 is designed to be best suited for PGY 2 residents.

Case Objectives: please list specific objectives for each of the domains you have checked above:

1. To identify whether residents address elements of shared decision making during TOLAC counseling

| SETTING: outpatient, in patient, ED, home, nursing home, rehab, group etc. | Outpatient setting |
| --- | --- |
| PATIENT PROFILE: Information about the “patient” that helps select an SP and helps the learner get an understanding of them as a person. SP will know more information about the patient than learner will ever ask but allows SP to portray a fully developed patient personality. If none of the items below are particulars for the case please write “all may be used.” | |
| Age range | 39 years |
| Religious/spiritual background | N/A |
| Sex (e.g., male, female, intersex, transwoman, transman) | Female |
| Sexual Orientation (e.g., heterosexual, lesbian, gay, bisexual, pansexual, queer, asexual) | Heterosexual |
| Gender expression (e.g., man, woman, gender queer) | Woman |
| Race/ethnicity: | African American |
| Physical description (e.g., BMI, height range) | In third trimester of pregnancy |
| Physical limitations | None |
| Patient appearance (e.g., disheveled, hospital gown, business casual, casual) | Casual clothing |
| Moulage + location (e.g., none, bruises, scars, body piercing, tattoos) | None |
| Affect (e.g., pleasant, cooperative) | Have mixed feelings about VBAC, but will do whatever the doctor suggests would be safest for you and the baby. |
| Family group (e.g., who is family, who they live with) | Lives with her 4 year old daughter and fiancé. Limited family in the area (just a cousin). |
| Education | N/A |
| Level of health literacy | N/A |
| Employment, if any - present and past, noting any current stresses | N/A |
| Home/homeless - type of dwelling, number of stories, owned or rented | N/A |
| Financial situation- any current stresses | N/A |
| Insurance Status (e.g., un/under/insured, public/private, HMO/PPO) | N/A |
| Habits (i.e., diet, exercise, caffeine, smoking, alcohol, drugs) | No smoking, tobacco, or drug use. |
| Activities (i.e., hobbies, sports, clubs, friends) | N/A |
| Typical day - what is the usual daily routine | N/A |

| CASE INFORMATION | |
| --- | --- |
| Chief Concern: What the patient will say when greeted by the student. The patient’s primary reason for seeking medical care often stated in his/own words. | You have a history of one prior C-section and are receiving prenatal care from a nurse practitioner at a community health center, who referred you to the Ob/Gyn for VBAC (vaginal birth after cesarean) counseling. |
| Additional Concerns: Other, if any, concerns the patient has today (i.e., symptoms, requests, expectations, etc.) that will become part of set agenda. | Planning for this to be your last child. Signed ‘tubal papers’ earlier during the pregnancy and plans for tubal ligation (tubes tied). |
|  | |
| THE PATIENT STORY: The SP will be asked to tell their symptom story and the personal and emotion impact for each of their concerns. You will want to write this is the patient voice. The symptom story should be able to answer this question: “Tell me more about [chief concern/additional concern], starting at the beginning and bringing me up to now.”  The personal context should be able to answer questions concerning the broader personal/psychosocial context of symptoms, especially the patient beliefs/attributions.  The emotional context should be able to ask how are you doing with this, how does this make you feel, how has this affected you emotionally? IMPACT: How has this affected your life? How has this been for your family? | **Instructions to SP:**  You have mixed feelings about VBAC and want more information before making a decision. You heard that VBAC is “dangerous” which, of course, worries you. Your coworker said she almost lost her baby trying to do it. But you remember how much easier it was to recover with your first baby, and now you will have three kids to care for with limited family support. On the other hand, it would be easier to make arrangements for childcare if you could schedule your delivery in advance, so the idea of a 39-week scheduled C-section is appealing. Overall, you would do whatever the doctor said, or seemed to suggest, was safest for you and the baby.  You are 39 years old and 35+4 weeks pregnant. This is your second pregnancy (due date is June 11th). You are not experiencing any contractions, bleeding, or leaking fluid. You feel the baby move on a regular basis.  Obstetrical History: You have had an uncomplicated pregnancy. Your first pregnancy was 4 years ago. You were induced at 41 weeks *“because I was overdue.”* You are not sure exactly how you were induced, but you do remember getting Pitocin. After an induction that lasted almost 2 days, you were completely dilated, but unable to push the baby out after trying for almost 3 hours. You ended up being delivered by C-section. The baby weighed 8 lbs 2 oz. Your postoperative course was complicated by a wound infection that required packing and extra office visits. It was a hassle that you would rather avoid if possible. You feel like you were so close, and with a little more time or effort, you think you could have done it. You would like another chance.  **Based on the information you are given and the interaction with the MD, you can choose whichever route of delivery you find more persuasive. If the pros and cons seem completely balanced, you can ask if it’s okay to talk it over with the baby’s father and then decide.**  **Opening statement:** *“I wanted to find out more about ‘VBAC.’ The nurse at my clinic said it’s safe, but I’ve heard mixed reviews.”* |
| HISTORY OF PRESENT ILLNESS: Although some of the HPI will be given in the patient’s symptom story, the learners will expand the story during the direct question section. Below describe the detailed history, usually about the chief concern, which the student must develop in order to make a useful assessment of the problem: | |
|  | |
| Onset (when; gradual or sudden) | None – You are not experiencing any contractions, bleeding, or leaking fluid. You feel the baby move on a regular basis. |
| Setting (what was going on or where was patient when symptoms first noticed?) | This is a consultation for TOLAC |
| Duration (how long) | N/A |
| Time relationships (frequency, constant or intermittent) | N/A |
| Location | N/A |
| Radiation | N/A |
| Quality | N/A |
| Amount | N/A |
| Aggravated by what | N/A |
| Relieved by what | N/A |
| Associated with what | N/A |
| Attitude (what does the patient think is the problem, and how does he/she feel about it) | You have mixed feelings about VBAC and want to get some more information about it before making a decision. |
| Overall course | N/A |
| REVIEW OF SYSTEMS: Significant positives and negatives | |
|  | Constipation |
|  |  |
|  |  |
|  |  |
|  | |
| Past medical history | None |
| Medication allergies (Name and reaction) | None |
| Environmental allergies (Name and reaction) | None |
| Illnesses | None |
| Vaccinations | N/A |
| Surgeries | C-Section with first pregnancy |
| Accidents/ injuries/ trauma | N/A |
| Hospitalization | N/A |
|  | |
| Inclusive sexual and reproductive history | |
| Sexual practices  Sexual partners  Protection: Use of safer sex practices  Use of birth control if appropriate  Risk of intimate partner violence | N/A |
| Ob/GYN HISTORY | 2 pregnancies total = 1 live birth & 1 current pregnancy |
| Medications | Prenatal vitamins, antacids for heartburn, stool softeners |
| Immunizations | N/A |
| Tobacco products: | Never |
| Alcohol | Denies |
| Drugs | Denies |
| Diet (describe) | N/A |
| Exercise (describe) | N/A |
| List any other important social history or information important to this case | Lives with her 4 year old daughter and fiancé. Limited family in the area (just a cousin). Planning for this to be your last child. Signed ‘tubal papers’ earlier during the pregnancy and plans for tubal ligation (tubes tied). |
| Family history |  |
| Mother, Father, Siblings, Grandparents, and other significant findings. | High blood pressure and high cholesterol in mom and dad |
|  |  |
| Physical Exam-  Note: Exam will not be performed for this case. MDs will be informed the following: Normal vitals, gravid abdomen, fundal height of 37, baby head down by abdominal exam. Fetal Heart Rate 140s | |
| PHYSICAL EXAM FINDINGS |  |
| 1. Written in layman’s terms | N/A |
| 1. General appearance- affect, appearance, position of patient at opening (i.e. sitting, laying down, holding abdomen etc.) | N/A |
| 1. Vital signs | N/A |
| 1. Specific findings and affect | N/A |
| 1. Response to certain physical movements | N/A |
|  |  |
| DIAGNOSIS AND DIFFERENTIAL |  |
| Diagnosis with support from positive and negative history and PE findings | N/A |
| Differential with support from positive and negative history and PE findings | N/A |
|  |  |
| MANAGEMENT OR DIAGNOSITIC PLAN | N/A |
|  |  |
| PROFESSIONALISM ISSUES OR CHALLENGES: | None |
