# Supplementary material for: Evaluating Shared Decision Making in Trial of Labor After Cesarean Counseling Using Objective Structured Clinical Examinations
Source: MedEdPORTAL. 2020 Mar 20;16:10891. doi: 10.15766/mep_2374-8265.10891 (PMC7182044; doi:10.15766/mep_2374-8265.10891)
Supplement: Supplementary file 1 — A. Case 1 SP Development Tool.docx B. Case 2 SP Development Tool.docx C. Case 3 SP Development Tool.docx D. Case 1 Door Note.docx E. Case 2 Door Note.docx F. Case 3 Door Note.docx G. Scoring Rubric.docx [file mep-16-10891-s001.zip › C. Case 3 SP Development Tool.docx]

Appendix C: *MedEdPORTAL* Standardized Patient Case Development Tool

Date: 2014 (academic year)

Primary Case Author: Brownsyne Tucker Edmonds, MD, MPH, MS

Secondary Case Author: N/A

Standardized Patient Educator: Not available – Our University’s simulation center employs trained standardized patients who provide support for all simulation training efforts conducted.

Name of Case: TOLAC OSCE Counseling Case 3

Name of educational and or assessment activity: TOLAC OSCE Counseling

Patient Name: Brenda Washington

Chief Complaint: Referred for VBAC consultation

Most likely Diagnosis and Differential with rationale from history and/or physical exam: N/A

Challenge question: N/A

Domains: Check all that apply

- Professionalism

X Communication and Interpersonal skills

- Medical History
- Physical exam

X Shared Decision Making

- Patient Education
- Clinical Reasoning
- Documentation
- Handoff
- Presentation
- Other:

Type and level of learner: Case 2 is designed to be best suited for PGY 2 residents.

Case Objectives: please list specific objectives for each of the domains you have checked above:

1. To identify whether residents address elements of shared decision making during TOLAC counseling

| SETTING: outpatient, in patient, ED, home, nursing home, rehab, group etc. | Outpatient setting |
| --- | --- |
| PATIENT PROFILE: Information about the “patient” that helps select an SP and helps the learner get an understanding of them as a person. SP will know more information about the patient than learner will ever ask but allows SP to portray a fully developed patient personality. If none of the items below are particulars for the case please write “all may be used.” | |
| Age range | 29 years |
| Religious/spiritual background | N/A |
| Sex (e.g., male, female, intersex, transwoman, transman) | Female |
| Sexual Orientation (e.g., heterosexual, lesbian, gay, bisexual, pansexual, queer, asexual) | Heterosexual |
| Gender expression (e.g., man, woman, gender queer) | Woman |
| Race/ethnicity: | African American |
| Physical description (e.g., BMI, height range) | In third trimester of pregnancy |
| Physical limitations | None |
| Patient appearance (e.g., disheveled, hospital gown, business casual, casual) | Casual clothing |
| Moulage + location (e.g., none, bruises, scars, body piercing, tattoos) | None |
| Affect (e.g., pleasant, cooperative) | You strongly desire to have a chance at a vaginal delivery. You feel strongly about your decision, and, based on your previous experiences with OBs, you are not sure if they are making their ‘recommendations’ for your good or for their own convenience. You are a little suspicious and possibly defensive, but NOT combative. You are just firm in your decision to attempt vaginal delivery. |
| Family group (e.g., who is family, who they live with) | Lives with her husband and 2 daughters (ages 4 and 2). Safe at home. Has family in the area (mom, sisters, nieces/nephews). |
| Education | N/A |
| Level of health literacy | N/A |
| Employment, if any - present and past, noting any current stresses | N/A |
| Home/homeless - type of dwelling, number of stories, owned or rented | N/A |
| Financial situation- any current stresses | N/A |
| Insurance Status (e.g., un/under/insured, public/private, HMO/PPO) | N/A |
| Habits (i.e., diet, exercise, caffeine, smoking, alcohol, drugs) | No smoking, tobacco, or drug use. |
| Activities (i.e., hobbies, sports, clubs, friends) | N/A |
| Typical day - what is the usual daily routine | N/A |

| CASE INFORMATION | |
| --- | --- |
| Chief Concern: What the patient will say when greeted by the student. The patient’s primary reason for seeking medical care often stated in his/own words. | You have a history of 2 prior C-sections and desire to attempt VBAC. You are receiving prenatal care from a nurse practitioner (NP) at one of the community health centers who referred you to Ob/Gyn for VBAC (vaginal birth after cesarean) counseling. |
| Additional Concerns: Other, if any, concerns the patient has today (i.e., symptoms, requests, expectations, etc.) that will become part of set agenda. | Planning for this to be your last child. Plans for tubal ligation. |
|  | |
| THE PATIENT STORY: The SP will be asked to tell their symptom story and the personal and emotion impact for each of their concerns. You will want to write this is the patient voice. The symptom story should be able to answer this question: “Tell me more about [chief concern/additional concern], starting at the beginning and bringing me up to now.”  The personal context should be able to answer questions concerning the broader personal/psychosocial context of symptoms, especially the patient beliefs/attributions.  The emotional context should be able to ask how are you doing with this, how does this make you feel, how has this affected you emotionally? IMPACT: How has this affected your life? How has this been for your family? | **Instructions to SP:**  You strongly desire to have a chance at a vaginal delivery. You feel like you were ‘robbed’ of the opportunity with both of your previous deliveries--you were ‘rushed’ with your first delivery and they ‘missed’ the breech presentation with your second delivery (see HPI). You’ve been told that VBAC is “dangerous” but believe that those are just scare tactics. You read on the internet that VBAC is in fact an option for women who have had two prior C-sections and your NP told you that it’s permitted, so you do not buy the ‘safety’ claims. You really hated having C-sections. You weren’t able to have your mom and sister in the room for either of the other deliveries; you hate that they take the baby away, and you’re left to worry and wonder if they are okay. Your husband’s never been able to cut the cord. And the recovery period was awful. You got a horrible headache from the needle they put in your back (spinal anesthesia). And the recovery takes so long. You have 2 other kids and you do not have 6 weeks to lay around or ‘take it easy’. You dislike the way the narcotic pain medications make you feel and itch.  This pregnancy, you did your research and you know your ‘rights.’ This is your last chance to have a vaginal delivery (planning to get your tubes tied), and it is something you have always wanted to experience. You want the skin to skin, the cord cutting, the immediate breastfeeding—the “Baby Story” experience. You feel strongly about your decision, and, based on your previous experiences with OBs, you are not sure if they are making their ‘recommendations’ for your good or for their own convenience. You are a little suspicious and possibly defensive, but NOT combative. You are just firm in your decision to attempt vaginal delivery.  You are 29 years old and currently 35+4 weeks pregnant. This is your third pregnancy (due date is May 24^th^). You are not experiencing any contractions, bleeding, or leaking fluid. You are feeling the baby move on a regular basis.  Obstetrical History: You have had an uncomplicated pregnancy. Your first pregnancy was 4 years ago. You were induced at 41 weeks *“because I was overdue.”* You are not sure exactly how you were induced, but you do remember they put some tablets in your vagina to start labor. After about 24 hours you were told you’d had a “failed induction” because you had not dilated, so you would have to be delivered by C-section. The baby weighed 6 lbs 4 oz. *“They didn’t even give me a chance! I know I could have delivered a 6 lb baby! I have friends who were in labor way longer than 24 hours. I think my doctors were just too impatient.”* You had planned to attempt VBAC with your second pregnancy, but when you came in laboring (at 40 weeks—on your due date), they realized the baby was butt down, so you had to have an urgent C-section (weighed 7lbs). You were really devastated by that, and a little bit angry with your doctors. *“Don’t you think someone should have picked up on that (breech) beforehand?”* This is your last chance to have a vaginal delivery, because you are planning to get your tubes tied, and it is something you have always wanted to experience. *“Don’t tell me it’s not allowed. I’ve read about it online. I know people do it and I know doctors can offer it.”*  If asked: You are unsure about epidurals. Have had to have 2 *“needles in my back”* with the other deliveries and got an awful headache after one of them. Regarding size of the baby--this baby feels like its somewhere in between the first two in size.  **Opening statement:** *“I want to VBAC. The nurse at my clinic said it’s allowed, but I’d need to have an obstetrician perform the delivery.”* |
| HISTORY OF PRESENT ILLNESS: Although some of the HPI will be given in the patient’s symptom story, the learners will expand the story during the direct question section. Below describe the detailed history, usually about the chief concern, which the student must develop in order to make a useful assessment of the problem: | |
|  | |
| Onset (when; gradual or sudden) | None – You are not experiencing any contractions, bleeding, or leaking fluid. You are feeling the baby move on a regular basis. |
| Setting (what was going on or where was patient when symptoms first noticed?) | This is a consultation for TOLAC |
| Duration (how long) | N/A |
| Time relationships (frequency, constant or intermittent) | N/A |
| Location | N/A |
| Radiation | N/A |
| Quality | N/A |
| Amount | N/A |
| Aggravated by what | N/A |
| Relieved by what | N/A |
| Associated with what | N/A |
| Attitude (what does the patient think is the problem, and how does he/she feel about it) | You feel strongly about your decision. You are a little suspicious and possibly defensive, but NOT combative. You are just firm in your decision to attempt vaginal delivery. |
| Overall course | N/A |
| REVIEW OF SYSTEMS: Significant positives and negatives | |
|  | Constipation |
|  |  |
|  |  |
|  |  |
|  | |
| Past medical history | None |
| Medication allergies (Name and reaction) | None |
| Environmental allergies (Name and reaction) | None |
| Illnesses | None |
| Vaccinations | N/A |
| Surgeries | C-Section with first and second pregnancies |
| Accidents/ injuries/ trauma | N/A |
| Hospitalization | N/A |
|  | |
| Inclusive sexual and reproductive history | |
| Sexual practices  Sexual partners  Protection: Use of safer sex practices  Use of birth control if appropriate  Risk of intimate partner violence | N/A |
| Ob/GYN HISTORY | 3 pregnancies total = 2 live births & 1 current pregnancy |
| Medications | Prenatal vitamins, antacids for heartburn, stool softeners |
| Immunizations | N/A |
| Tobacco products: | Never |
| Alcohol | Denies |
| Drugs | Denies |
| Diet (describe) | N/A |
| Exercise (describe) | N/A |
| List any other important social history or information important to this case | Lives with her husband and 2 daughters (ages 4 and 2). Safe at home. Has family in the area (mom, sisters, nieces/nephews). Plans for tubal ligation. |
| Family history |  |
| Mother, Father, Siblings, Grandparents, and other significant findings. | High blood pressure and high cholesterol in mom and dad |
|  |  |
| Physical Exam-  Note: Exam will not be performed for this case. MDs will be informed the following: Normal vitals, gravid abdomen, fundal height of 36, baby head down by abdominal exam. Fetal Heart Rate 140s | |
| PHYSICAL EXAM FINDINGS |  |
| 1. Written in layman’s terms | N/A |
| 1. General appearance- affect, appearance, position of patient at opening (i.e. sitting, laying down, holding abdomen etc.) | N/A |
| 1. Vital signs | N/A |
| 1. Specific findings and affect | N/A |
| 1. Response to certain physical movements | N/A |
|  |  |
| DIAGNOSIS AND DIFFERENTIAL |  |
| Diagnosis with support from positive and negative history and PE findings | N/A |
| Differential with support from positive and negative history and PE findings | N/A |
|  |  |
| MANAGEMENT OR DIAGNOSITIC PLAN | N/A |
|  |  |
| PROFESSIONALISM ISSUES OR CHALLENGES: | None |
