# Supplementary material for: Evaluating Shared Decision Making in Trial of Labor After Cesarean Counseling Using Objective Structured Clinical Examinations
Source: MedEdPORTAL. 2020 Mar 20;16:10891. doi: 10.15766/mep_2374-8265.10891 (PMC7182044; doi:10.15766/mep_2374-8265.10891)
Supplement: Supplementary file 1 — A. Case 1 SP Development Tool.docx B. Case 2 SP Development Tool.docx C. Case 3 SP Development Tool.docx D. Case 1 Door Note.docx E. Case 2 Door Note.docx F. Case 3 Door Note.docx G. Scoring Rubric.docx [file mep-16-10891-s001.zip › E. Case 2 Door Note.docx]

**Appendix E. TOLAC Counseling OSCE Case 2**

**‘MD-Facing’ Door Note/Chart/Report**

(Resident Directions Prior to Encounter)

**Patient Name**: Brenda Washington

**Setting:** Outpatient clinic

**Complaint**: Referred for Vaginal birth after cesarean section consultation.

**HPI:** Ms. Washington is a 39 year old G2P1001 @ 35/4 by 16 week ultrasound who has been receiving prenatal care from a nurse practitioner at a local community health center. She was referred to your clinic for a vaginal birth after cesarean section consultation. Her first pregnancy resulted in a primary low-transverse cesarean section due to arrest of descent at 41/2 weeks following a postdates induction. Her operative note confirms low-transverse cesarean section. Her medical, surgical, and family histories are otherwise unremarkable. She denies smoking, alcohol and drug use. She lives with her daughter and fiancé. Her review of systems is noncontributory and her exam findings are below.

**Exam**:

| Temperature: 98.2 | Respiratory Rate: 24 | Blood Pressure: 121/73 | Heart Rate: 83 |
| --- | --- | --- | --- |
| Height: 5’ 4’’ | Weight: 175 lb | Body Mass Index: 30 |  |
| Fundal Height: 37 | Fetal Heart Rate: 140s | Estimated Fetal Weight: 3704g (92%ile) | Vertex |

*You have_15___ minutes to counsel the patient as you would in your typical practice and develop a plan of care for her delivery.*

*After you are finished with your patient, please exit the room and write a note to document your discussion.*
