# Supplementary material for: Evaluating Shared Decision Making in Trial of Labor After Cesarean Counseling Using Objective Structured Clinical Examinations
Source: MedEdPORTAL. 2020 Mar 20;16:10891. doi: 10.15766/mep_2374-8265.10891 (PMC7182044; doi:10.15766/mep_2374-8265.10891)
Supplement: Supplementary file 1 — A. Case 1 SP Development Tool.docx B. Case 2 SP Development Tool.docx C. Case 3 SP Development Tool.docx D. Case 1 Door Note.docx E. Case 2 Door Note.docx F. Case 3 Door Note.docx G. Scoring Rubric.docx [file mep-16-10891-s001.zip › F. Case 3 Door Note.docx]

**Appendix F. TOLAC Counseling OSCE Case 3**

**‘MD-Facing’ Door Note/Chart/Report**

(Resident Directions Prior to Encounter)

**Patient Name**: Brenda Washington

**Setting:** Outpatient clinic

**Complaint**: Referred for Vaginal birth after cesarean section consultation.

**HPI:** Ms. Washington is a 29 year old G3P2002 @ 35/4 by 16 week ultrasound who has been receiving prenatal care from a nurse practitioner at a local community health center. She was referred to your clinic for a vaginal birth after cesarean section consultation. Her first pregnancy was uncomplicated, but resulted in a failed postdates induction and primary low-transverse cesarean section at 40+1. Her second pregnancy resulted in spontaneous labor at 40 weeks, but required repeat cesarean delivery due to malpresentation noted in triage. Her pelvis is proven to 7 lb 0 oz. Her operative notes confirm low-transverse cesarean section. Her medical, surgical, and family histories are otherwise unremarkable. She denies smoking, alcohol and drug use. She lives with her husband and 2 daughters. Her review of systems is noncontributory and her exam findings are below.

**Exam**:

| Temperature: 98.2 | Respiratory Rate: 24 | Blood Pressure: 121/73 | Heart Rate: 83 |
| --- | --- | --- | --- |
| Height: 5’ 6’’ | Weight: 154 lb | Body Mass Index: 25 |  |
| Fundal Height: 36 | Vertex by Leopold’s | Fetal Heart Rate: 140s |  |

*You have_15_ minutes to counsel the patient as you would in your typical practice and develop a plan of care for her delivery.*

*After you are finished with your patient, please exit the room and write a note to document your discussion.*
