# Supplementary material for: Evaluating Shared Decision Making in Trial of Labor After Cesarean Counseling Using Objective Structured Clinical Examinations
Source: MedEdPORTAL. 2020 Mar 20;16:10891. doi: 10.15766/mep_2374-8265.10891 (PMC7182044; doi:10.15766/mep_2374-8265.10891)
Supplement: Supplementary file 1 — A. Case 1 SP Development Tool.docx B. Case 2 SP Development Tool.docx C. Case 3 SP Development Tool.docx D. Case 1 Door Note.docx E. Case 2 Door Note.docx F. Case 3 Door Note.docx G. Scoring Rubric.docx [file mep-16-10891-s001.zip › G. Scoring Rubric.docx]

**Appendix D. TOLAC Counseling OSCE**

**Resident Scoring Rubric Form**

1. **Resident ID ____________________ Coder Initials ______________ Date of coding ___________**

**Elements of Informed Decision Making Ratings^a^**:

| **Element** | **Accomplished** | **Rating Notes** | **Examples** |
| --- | --- | --- | --- |
| 1. Does the resident discuss the clinical issue or nature of the decision (e.g. explain TOLAC, VBAC, ERCD to the patient) | absent partial complete  0 1 2 | **Absent – MD does not explain what TOLAC vs VBAC entails and does not indicate that the patient must choose between an ERCD and TOLAC.** | No explanation |
|  |  | **Partial** – MD explains what a TOLAC vs VBAC entails OR indicates that the patient must choose between an ERCD and TOLAC (not both). | *“For women that have had one or two prior cesarean deliveries, there are a few options for mode of delivery. Some women prefer to have a scheduled, repeat cesarean section; while others would prefer vaginal birth after cesarean, or VBAC. For women that prefer a vaginal delivery, they may choose to attempt a trial of labor after cesarean or TOLAC.*  OR  *“There are risks and benefits to each approach, so we leave it to you to choose.”* |
|  |  | **Complete** – MD explains what a TOLAC vs VBAC entails AND indicates that the patient must choose between an ERCD and TOLAC. | *“For women that have had one or two prior cesarean deliveries, there are a few options for mode of delivery. Some women prefer to have a scheduled, repeat cesarean section; while others would prefer vaginal birth after cesarean, or VBAC. For women that prefer a vaginal delivery, they may choose to attempt a trial of labor after cesarean or TOLAC. There are risks and benefits to each approach, so we leave it to you to choose.”* |
| 2. Does the resident inform the patient of her ‘prognosis’ (chance of successful VBAC vs failure requiring RCD)? | absent partial complete  0 1 2 | **Absent** – MD does not explain patient’s likelihood of successful VBAC vs failure requiring RCD | No explanation |
|  |  | **Partial** – MD explains the patient’s likelihood of successful VBAC but does not mention failure requiring RCD | *“Based on your clinical characteristics, such as your history of prior vaginal deliveries, it is likely that you would have a successful VBAC.”* |
|  |  | **Complete** – MD explains the patient’s likelihood of successful VBAC AND explains the possibility of failure requiring RCD | *“Based on your clinical characteristics, such as your history of prior vaginal deliveries, it is likely that you would have a successful VBAC; however, it is important for you to know that if the TOLAC fails, we must proceed to a RCD and that has potential to be an urgent/emergent situation.”* |
| 3. Does the resident discuss the alternatives modes of delivery/outcomes.  a. successful TOLAC    b. failed TOLAC with RCD  c. scheduled ERCD  d. other (specify under notes) | absent partial complete  0 1 2  absent partial complete  0 1 2  absent partial complete  0 1 2  absent partial complete  0 1 2 | **Absent** – MD does not address alternatives or MD dictates management plan. | No explanation |
|  |  | **Partial** – MD discusses options but does not describe potential range of outcomes for VBAC. Must be an explicit statement of the alternatives (not just an implicit understanding that there are only two ways out) | *“You have two options for mode of delivery. Vaginal birth or ERCD ”* |
|  |  | **Complete** – MD explains both management strategies AND describes associated outcomes | *“You have two options for mode of delivery. Vaginal birth or ERCD. It is important for you to know that if you choose to have a vaginal birth there are a few different outcomes that you may encounter:*  *1. TOLAC that results in a successful VBAC*  *2. Failed TOLAC that results in a RCD”* |
| 4a. Does the resident discuss risks to baby?  i. TOLAC    ii. TOLAC+RCD  iii. scheduled ERCD  iv. other (specified above) | absent partial complete  0 1 2  absent partial complete  0 1 2  absent partial complete  0 1 2  absent partial complete  0 1 2 | Absent – No mention of risks to baby associated with the modes of delivery presented | No risks mentioned |
|  |  | Partial – MD discusses risks to baby related to TOLAC OR ERCD (PLEASE NOTE WHICH ONES) | Examples:  **TOLAC**   - Dystocia - Brain injury   **RCD**   - Respiratory distress - Risk of laceration during surgery - Prematurity - Low birth weight |
|  |  | Complete MD discusses risks to baby related to TOLAC AND ERCD. (PLEASE NOTE IF EXPLICIT COMMENT MADE ON INCREASED MORBIDITY ASSOCIATED WITH FAILED VBAC) |  |
| 4b. Does the resident discuss risks to mom?  i. TOLAC    ii. TOLAC+RCD  iii. scheduled ERCD  iv. other (specified above) | absent partial complete  0 1 2  absent partial complete  0 1 2  absent partial complete  0 1 2  absent partial complete  0 1 2 | **Absent** – No mention of risks to mom associated with the modes of delivery presented | No risks mentioned |
|  |  | **Partial** – MD discusses risks to mom related to TOLAC OR ERCD (PLEASE NOTE WHICH ONES) | Examples:  **TOLAC**   - Rupture or cesarean scar or uterine rupture - Possible tearing or episiotomy   **TOLAC + RCD**   - Risk of infection doubles if vaginal delivery is attempted but results in cesarean, morbidity associated with failed VBAC   **RCD**   - Usual risks of surgery (bleeding, infection, injury to surrounding organs, hysterectomy) - Ongoing pain and discomfort at incision - Increased risk with multiple CDs |
|  |  | **Complete** – MD discusses risks to mom related to TOLAC AND ERCD. (PLEASE NOTE IF EXPLICIT COMMENT MADE ON INCREASED MORBIDITY ASSOCIATED WITH FAILED VBAC) |  |
| 5a. Does the resident discuss benefits to baby?  i. TOLAC    ii. TOLAC+RCD  iii. scheduled ERCD  iv. other (specified above) | absent partial complete  0 1 2  absent partial complete  0 1 2  absent partial complete  0 1 2  absent partial complete  0 1 2 | **Absent** – No mention of benefit to baby associated with the modes of delivery presented | No benefits mentioned |
|  |  | **Partial** – MD discusses benefits to baby related to TOLAC OR ERCD (PLEASE NOTE WHICH ONES) | Examples:  **TOLAC**   - Lungs clear as baby passes through birth canal - Facilitates bonding and breastfeeding   **RCD**   - Scheduled procedure vs. emergency surgery |
|  |  | **Complete** – MD discusses benefits to baby related to TOLAC AND ERCD. |  |
| 5b. Does the resident discuss benefits to mom?  i. TOLAC    ii. TOLAC+RCD  iii. scheduled ERCD  iv. other (specified above) | absent partial complete  0 1 2  absent partial complete  0 1 2  absent partial complete  0 1 2  absent partial complete  0 1 2 | **Absent** – No mention of benefit to mom associated with the modes of delivery presented | No benefits mentioned |
|  |  | **Partial** – MD discusses benefits to mom related to TOLAC OR ERCD (PLEASE NOTE WHICH ONES) | Examples:  TOLAC   - No abdominal surgery - Shorter recovery period - Lower risk of infection - Less blood loss - Temporary vaginal pain - Shorter hospital stay - Cord cutting - Family involvement - Skin to skin   TOLAC + RCD   - Emergency surgery - Greater blood loss - Right of infections   RCD   - Scheduled procedure vs emergency surgery |
|  |  | **Complete** – MD discusses benefits to mom related to TOLAC AND ERCD |  |
| 6. Does the resident discuss the patient’s goal/context of decision  --Desired birth experience  --Competing priorities  --Risk aversion | absent partial complete  0 1 2 | **Absent** – No effort to elicit patient’s concerns, goals, or factors that may impact the patient’s decision-making | No eliciting of patient concerns. |
|  |  | **Partial** – MD asks about at least 1 factor OR consideration influencing their mode of delivery preference | *“What is your desired birth experience?”*  *“What is most important to you in making this decision?”* |
|  |  | **Complete** – MD asks about 2 or more factors or considerations influencing their mode of delivery preference AND inquiries about their risk aversion | *“In making a decision like this, it is often important to determine how risk averse you are? As in, are you someone that prefers a more certain outcome?”* |
| 7. Does the resident discuss the uncertainties associated with the decision?  --Do they address ‘unknowns’ such as spontaneous labor, inability to predict who will rupture, limits of predictions of success | absent partial complete  0 1 2 | **Absent** – No mention of uncertainty related to spontaneous labor/rupture or limits of predicted likelihood of success | No discussion of uncertainty. |
|  |  | **Partial** – MD mentions uncertainty related to spontaneous labor/rupture OR limits of predicted likelihood of success | *“It is difficult for us to predict when someone will rupture and go into labor.”*  OR  *“There are several important factors that contribute to your likelihood of a successful VBAC and it is difficult for us to accurately predict whether or not you will be successful.”* |
|  |  | **Complete** – MD mentions uncertainty related to spontaneous labor/rupture AND limits of predicted likelihood of success | *“If you go into labor on your own, you're much more likely to be successful with a trial of labor after cesarean section, and your risk of the breaking open of the uterus or the uterine rupture is lower…* *another approach is to say if my body's ready…to go into labor on my own, we'll go for a vaginal delivery. If not, then we'll go with a C-section.”* |
| 8. Does the resident discuss (or designate) the patient’s role in decision making?  --Do they specify who should/would make that decision re: MOD? | absent partial complete  0 1 2 | **Absent** – MD does not explicitly state the patient has a role in the decision-making process. | *“It is important to have a plan for mode of delivery.”* |
|  |  | **Partial** – MD indicates the patient has a role | *“It is my job to make sure you know all of your options so that you can make an informed decision.”* |
|  |  | **Complete** – MD indicates a SHARED role | *“This is a decision you should make based on what you believe is best for you and your baby. I’m here to help you make that decision.”* |
| 9. Does the resident assess the patient’s understanding?  --Ask-Tell-Ask, Invite questions, Teach back, | absent partial complete  0 1 2 | **Absent** – MD does not assess patient understanding | *“I hope this information was helpful.”* |
|  |  | **Partial** – MD inquires as to whether the patient understands | *“Do you have any questions about the options we discussed?”* |
|  |  | **Complete** – MD ask patient to demonstrate their understanding. (Teach-back or ASK-Tell Ask required) | *“Tell me what you understand about the options we discussed.”* |
| 10. Does the resident explore the patient’s preference (based on the conversation, which way is the patient leaning?) | absent partial complete  0 1 2 | **Absent** – No mention of need for decision or inquiry about preference | *“I will let you think about your options.”* |
|  |  | **Partial** – MD defers discussion of decision/preference | *“Do you need time to think about your options? You do not have to make a decision today.”* |
|  |  | **Complete** – MD asks about preference (ok to defer if patient states they are unsure) | *“Do you have an idea of what you would prefer to do?”* |

**^a^This rubric was adapted from Braddock’s previously developed 9-item informed decision making scale.**

Braddock CH 3rd, Edwards KA, Hasenberg NM, Laidley TL, Levinson W. Informed decision making in outpatient practice: time to get back to basics. *JAMA.* Dec 22–29; 1999 282(24):2313– 2320.

Braddock C 3rd, Hudak PL, Feldman JJ, Bereknyei S, Frankel RM, Levinson W. “Surgery is certainly one good option”: quality and time-efficiency of informed decision-making in surgery. *J Bone Joint Surg Am.* 2008 Sep;90(9):1830-8.

Salyers MP, Matthias MS, Fukui S, et al. A coding system to measure elements of shared decision making during psychiatric visits. *Psychiatr Serv.* 2012 Aug;63(8):779-84.
